# How to measure mental illness stigma at work: development and validation of the workplace mental illness stigma scale

**DOI:** 10.3389/fpsyt.2023.1225838

**Published:** 2023-07-12

**Authors:** Naseli Matousian, Kathleen Otto

**Affiliations:** Department of Work and Organizational Psychology, Faculty of Psychology, Philipps University of Marburg, Marburg, Germany

**Keywords:** mental health stigma, workplace reintegration, attitudes, discrimination, mental health at the workplace, return-to-work

## Abstract

**Introduction:**

The study objective was to design a new theoretically driven multidimensional scale for the use in the empirical measurement of stigmatizing attitudes towards persons with mental illness within the return-to-work process as this integral part of vocational reintegration has been widely neglected by scholars so far.

**Methods:**

Therefore, we developed and validated a 21-item instrument to comprehensively measure the three-factorial structure of stigmatizing attitudes (affect, cognition, behavior) across two studies (overall N = 251).

**Results:**

In both studies the new scale proved to be highly internally consistent, and its proposed three-factor structure was equally supported across the two studies. Convergent and discriminant validity were demonstrated by moderate and high correlations or zero correlations with pertinent measures. Furthermore, construct validity of the new scale was supported by significant positive associations with relevant personality characteristics within stigma research.

**Discussion:**

The WMISS is the first instrument to measure mental health stigma specifically within the return-to-work-process and demonstrates strong psychometric properties. Inclusion of this scale in future research can help facilitate understanding of mental illness stigma within the occupational sector and assist with targeted intervention development.

## Mental illness stigma in the workplace

One of the most challenged societal sectors with respect to mental disorders and its stigmatization is the professional and working life. Specifically, the average level of sick leave due to a mental health problem amounts to 30 days per year in Germany, whereas diseases of the muscular-skeletal system entail about 18 days of incapacity to work (1). Moreover, since the 1990s the days of sick leave due to a physical illness have declined, while sickness absence and early retirements as a result of mental ill-health have continuously grown in Germany, a trend, which is also globally observable (2–4). These figures are especially worrisome since work plays an integral part in our lives. Gainful employment provides a daily routine, meaningful tasks and boosts self-confidence and enhances quality of life. Thus, work conveys a sense of self-worth and fosters societal integration and support (5). It contributes to economic prosperity, but also to personal and social fulfillment. Besides, work itself functions as a major source of mental health and is a socially integrating determinant which is highly valued. In addition, maintaining or returning to employment can be a key stepping-stone in the recovery process of employees who have suffered from poor mental health [e.g., (6–8)]. Precisely in such cases, it is of preeminent importance to ensure a safe and conducive working environment for people with mental health issues which promotes a considerate reintegration into the workforce. But unfortunately, mental health consumers still face a wide range of employment barriers and stigmatization as well as potential harassment within the workplace (9, 10). Past research has shown that unemployment rates among severely mentally ill individuals remain inordinately high, typically between 80 and 90% [e.g., (11–14)]. Concerns about the employability of individuals with mental illness were shown among the general population, as well as on the managerial and employee level (15).

In fact, a decent body of research on recruitment and employment of mental health consumers already exists: In a mixed methods design, Biggs et al. (16) reported, that while employment agencies would consider putting forward individuals with a mental health condition to employers, managers had reservations about hiring them due to mistrust and need of supervision. Surveys of US employers demonstrated that half of them were hesitant to hire someone with past psychiatric history or currently undergoing treatment for depression, with rates of reluctance to hire someone being even higher when the employee was said to have a history of substance abuse (17). In a nationwide interview-based assessment of mental health consumers in the United States concerning their experiences of stigmatization, Wahl (18) revealed that approximately one in three consumers had been turned down for a job for which they were qualified after their mental illness diagnosis was disclosed. This is concordant with recent results yielded from a field experiment, in which fictious applicants indicating a history of mental disorder received fewer callbacks than candidates with a history of physical injury (19). Compared to applicants with a physical condition, (20) showed that aspirants with a label of a depression disorder, had significantly less prospects of employment. Human resource managers responded notably biased for executive positions, 58% stating they would never employ someone with a diagnosis of depression for an executive job, compared to only 3% for a similar job candidate with diabetes. This stigmatization was grounded on the assumption of potential impaired work performance, rather than expectations of future absenteeism (20). Similar findings emanated from focus group interviews with 16 individuals with varying mental illnesses, showing that nearly all the participants concluded that their mental health condition had cost them previous job opportunities (21). Thus, disclosure of a mental illness in the labor market seems to cause direct detriment to the career possibilities of mental health patients, undermining their employability and jeopardizing career advancement (22, 23). Mental health consumers often prefer keeping their condition a secret, justifying long absences from work with fictional or fake diagnoses (24). Accordingly, they cannot then request rightful workplace accommodations which would ease their transition into the labor market and grant effective treatment.

But what happens when employees return to their workplace after a mental health diagnosis? Little attention has been paid to the return-to-work process of employees with mental disorders compared to people with physical disabilities (25). Yet, it seems that it is as difficult staying in the workforce as it is entering the labor pool, since mentally ill workers are confronted with a wide range of challenges and stigma, underemployment as being among the most pervasive obstacles. Underemployment refers to a situation in which individuals are forced to work in jobs inferior to their skills and qualifications or below adequate wages. Statistics indicate that 68% of workers with a mental health problem return to positions with reduced responsibility, work fewer hours and are paid less than before (26, 27). The annual income of employees with a depressive disorder is even decreased by approximately 10% compared to unaffected individuals (28). Additionally, mentally ill employees encounter interpersonal and social difficulties and discrimination, both at collegial and at executive level. They report about little or no psychosocial support and enhanced supervision (18, 24). Often, workers with a mental health condition become marginalized and targets for critical or negative remarks from workmates who had been previously helpful and accommodating (18, 24, 29).

Stereotypic beliefs about co-workers with mental health issues also influence attitudes about their performance capabilities and personality traits. Employees with depression and bipolar disorder were perceived to be low in warmth and competence, whereas workers with anxiety disorder were perceived as low in competence (30). Concerns about safety, incompetence and social compatibility with other co-workers were similarly identified as barriers to hiring and working with people with a mental health condition in a more recent interview-based study with employers and co-workers (31). These findings, too, dovetail well with qualitative research in the manifestations of stigma in the labor force. To that end, Russinova et al. (32) compiled an extensive taxonomy of prejudicial and discriminatory practices at the workplace gathered from a national sample of individuals with serious mental illnesses. Qualitative analyzes generated a continuum of more subtle to more blatant, but also anticipated, forms of psychiatric illness stigmatization experienced by mental health consumers that could be divided into two contextual domains: work performance and collegial interactions. Stigmatizing actions toward individuals with psychiatric conditions which do not aim at their work performance or job duties, but rather are enacted on an interactional-collegial level, appearing as similar to incivility, are especially toxic, since they erode one’s sense of identity and impede the integration into the social fabric of the working environment (33).

Further, stereotypic conceptions also affect how co-workers and employers feel about employees with mental illnesses. In a vignette-based study, participants who viewed the employee as responsible for his mental health condition, reacted to him in an angry way resulting in lesser approval of supported employment (34). Oftentimes, negative cognitions and feelings toward employees with a mental health condition precipitate discriminatory action. For example, feelings of fear toward individuals with mental illness predicted avoidance behaviors and stereotypic assumptions predicted work-related social distancing intentions (30, 34). These findings are consonant with data maintained from the United States Equal Employment Opportunity Commission (EEOC), supporting instances of formal and informal discrimination toward this employment population.

A review of charges filed under the Americans with Disabilities Act (ADA) found that the percentage of charges citing a psychiatric disability has increased over the period of 2005–2014 (10). Among these cases, harassment is cited more often on charges mentioning a psychiatric disability (22% of charges) as compared to ADA charges overall during this period (15%), highlighting that one of the most prominent conflict areas, which employees with a mental health condition encounter, are interpersonal-related stress factors with colleagues. Case studies of ADA charges revealed disability-specific mistreatment, such as being mandated to share information about a disability status beyond what is required for an accommodation request as well as enduring attempts of marginalization and isolation by taking away clients or setting meetings when the individual was unable to attend as well as references to their psychiatric disability in a disparaging and ridiculing manner (10). Also, job accommodations supporting employees with mental ill-health can be met by negative affective reactions from co-workers, perceiving the situation as unfair to themselves (35). Equally an expert study consulting mental illness-related labor and advocacy groups revealed that only 26.2% of experts indicated that employees could speak openly about mental health issues, and 81.5% of experts agreed that a large or medium unmet need for support for employees with mental health issues exists (36).

Taken together, it shows that employer and coworker attitudes are vital for beneficial workplace experiences for these employees and the success of reintegration programs, both the extent to which mentally ill employees are accepted into the occupational life as well as the extent to which equitable workplace accommodations are provided. Also, because mental health issues are one of the most common disabilities, it is essential to create a welcoming and inclusive workplace culture that may positively impact all employees, not just the ones with disabilities. Addressing negative attitudes and misconceptions about mental illnesses in the working environment would be the first step to foster successful mental illness literacy among the work force and establish an encouraging climate. In that respect, Corbiere et al. (37) classified presence of supportive colleagues, peer support networks, increased communication between the union and employees, and regular contact between employees and their company during sick leave as imperative elements for building an organizational culture supportive of successful return to work.

In light of the personal and economical costs to both organizations and individuals as well as the high prevalence of mental diseases globally, it is important that employees who are sick listed with mental health problems are facilitated in their return to work. In order to design valuable and tailor-made interventions, it is necessary to gather a better understanding of the reintegration process of people with mental health issues. Work-related stress factors such as mental illness stigmatization in the workplace might be especially deleterious within this crucial transitional process. To our knowledge, no instruments are available that capture mental illness stigma regarding the return-to-work process of employees with mental health problems. However, a measure that addresses and highlights stigmatizing attitudes toward people with mental health problems upon returning to work is not only relevant for empirical research but also of great use for the evaluation of the effects of workplace intervention programs.

## Mechanisms of stigma and assessment of stigma

The process of stigmatization itself is closely intertwined with the tripartite view of attitudinal research. In that sense attitude structures contain cognitive, affective, and behavioral components (38, 39). Stigmatizing attitudes are likewise in line with this affective-cognitive-behavioral framework originating from social-psychological conceptualizations and therefore encompass stereotypes, prejudices, and discrimination. Stereotypes are global cognitive knowledge representations, which consist of unfavorable and adverse assumptions, evaluations and opinions about a certain social group and are collectively applied to individuals of that group of people (40). These cognitive correlates of stigmatization usually involve characterizations of incompetence, dangerousness and weakness concerning one group compared to another. Prejudices, however, are defined as the negative emotional reactions toward certain social groups and their members resulting from stigmatizing attitudes. The negative affective responses can range from pity to fear up to annoyance and anger regarding the stigmatized group. They, in turn, can lead to patronizing, hostile and avoidant behavior toward this group of people, which is the discriminatory aspect of stigmatizing processes. Discriminatory practices comprise a broad spectrum of disadvantaging behavioral actions, such as differential treatment of one group relative to another by withholding assistance and support toward the stigmatized group and, in the worst case, restricting them from life opportunities and rights. Further, the existence of power imbalance plays a paramount role in situations, in which stigmatization occurs. Thus, social, economic, and political power is necessary to stigmatize putative deviant members of a society (41). Stigmatization, hence, involves the confluence of different interrelated dimensions - stereotype, prejudice, and discrimination–but also the occurrence of power inequality which allows stigmatizing attitudes to be applied and acted upon. It is important to identify and understand these different core features of public stigma to effectively design targeted empirical research and plan specific interventions for stigma reduction campaigns.

However, assessment of mental illness stigma is heterogenous in measurements as well as in theoretical underpinnings and has mostly centered around attitudes of the general population toward individuals with mental health issues in general. One of the first scales was the Opinions about Mental Illness (OMI) scale (42), developed further by Taylor and Dear (43), resulting in the Community Attitudes to Mental Illness Inventory (CAMI). The CAMI measures attitudes in the general public and encompasses 40 items covering four sub-scales on authoritarianism, benevolence, social restrictiveness and community mental health ideology. Psychometric analyzes yielded adequate results in various samples in the United States and Canada. The inventory has been widely employed and translated into several languages, including Spanish, Italian, and German (44–46). Most of the questionnaires in stigma research incorporate common cognitive statements and opinions about mental illnesses in general, either gaged through personal or perceived stigma in society [e.g. (47–49),]. As a result, emotional and affective responses of the stigmatizer toward individuals with mental health issues are currently underassessed in stigma research (50). The Emotional Reaction to Mental Illness Scale developed by Angermeyer and Matschinger (51) is one of few scales which explicitly assesses affective reactions toward persons with mental illnesses, utilizing two vignette descriptions, one depicting schizophrenia and the other major depression. The final version contains four items of the three dimensions aggressive emotions (e.g., anger, irritation), prosocial reactions (desire to help, sympathy) and feelings of anxiety (uneasiness, fear). Link (52) constructed a 12-items questionnaire which determines the respondent’s perceptions of what most other people believe regarding devaluation and discrimination of people with mental illnesses in job, friendships, and romantic relationships. The scale has been mainly administered among people with mental health issues but can also be applied in other populations. The 9-item short form of the AQ-27, the Attribution Questionnaire (AQ-27), was created by Corrigan et al. (53) by extracting 9 items from the AQ-27 with the highest factor loadings measuring the domains of blame, anger, pity, help, dangerousness, fear, avoidance, segregation, and coercion. In a series of studies Griffiths and her colleagues generated measurements for stigma concerning depression and general anxiety disorders, identifying statements specific to stigmatizing beliefs about these illnesses (54, 55). The Prejudice toward People with Mental Illness Scale (PPMI), in turn, focuses on the concept of prejudice as an antecedent of discriminatory behavior and pinpoints four underlying factors: fear/avoidance, malevolence, authoritarianism, and unpredictability (56). As the only instrument to date to assess microaggressions perpetrated toward persons with mental illness, the mental illness microaggressions scale (MIMS-P) comprises 17 items resulting in three sub-scales, referring to Assumption of Inferiority, Patronization and Fear of Mental Illness (57). Behavioral inclinations toward people with mental illness have most commonly been determined by using measures of social distance, which assess a respondent’s desire to interact with a target person in different forms of relationships [e.g., (30, 58); Link et al., 1999 (59)]. The vast majority of measures assessing mental health stigma represent global opinions about mental illnesses and do not cover specific life areas or social situations, such as the professional and occupational sector. An instrument to assess attitudes toward individuals with disabilities in the workplace was developed by Popovich et al. (60) consisting of scales regarding beliefs about what constitutes a disability, affective reactions to working with individuals with disabilities and opinions about the reasonableness of common workplace accommodations. The Workplace Inclusion Questionnaire (WIQ) specifically addresses attitudes regarding workplace inclusion which vary across different case stories including descriptions of individuals with musculoskeletal and mental disorders (61). Taken together, most stigma measures focus on a general stigmatization context, underrepresent specific mental disorders and lack of the affective component underlying the attitudinal framework.

## The present study

This research focuses on the multifaceted and multidimensional elements of mental illness stigma in the workplace. We want to provide a comprehensive examination of the various components that comprise stigma by creating a targeted multidimensional mental illness stigma scale in the workplace. To eliminate disadvantages and social misconceptions about individuals with mental ill-health in the vocational reintegration process, we must first have a clear picture of the structure and substance of stigmatizing attitudes toward people with mental health issues. Thus, the aim of this study was to close the aforementioned gap in workplace stigmatization research concerning the return-to-work process as well as to develop and validate a measure referring to mental illness stigma in the workplace within the cognitive-affective-behavioral scheme, assessing emotional reactions, cognitive structures and behavioral consequences, all resulting in stigmatizing attitudes. To that end, two studies were conducted: Study 1 focused on the measurement development including item generation and analyzes as well as exploratory factor and validity analyzes, Study 2 utilized confirmatory factor analysis to evaluate the fit of the measures and expand the knowledge on validity of the new scale.

## Study 1: item generation and content validation

Based on the three-pronged approach within the attitudinal framework and aiming to meet the requirement of multidimensionality as recommended by Antonak and Livneh (2000) (62) as well as in concordance with the multidimensional attitudes scale toward persons with disabilities (63), we represented each of the three dimensions of stigmatizing attitudes separately. Furthermore, in the development of our questionnaire, we incorporated items from established scales measuring different aspects of workplace attitudes toward various employment populations as well as items employed within mental health stigma research in order to generate an exhaustive image of workplace attitudes regarding mentally ill employees. This resulted in an initial item pool of 83 items.

### Methods

#### Participants

Data of *N* = 104 could be collected. Respondents ranged in age between 18 and 61, with an average age of 24.44 years (SD = 7.09). Over 60% of the participants were female and 55.7% had completed general qualification for university entrance. One participant did not state their age, and two respondents did not state their exposure to mental illness. Exclusion of these data was refrained from, as a systematic bias was not to be expected. With over 62%, the majority of the participants were college students. Overall, 72% reported they had experiences with mental illness.

#### Vignettes

The three subscales were preceded by a vignette depicting a scenario in the working environment to have participants project their own feelings, thoughts, and behavioral intentions onto the indicated situation. Vignette-design studies are one of the most common methodological techniques in stigma research and show wide-ranging applicability (50). Using this approach, we intended to provide an elaborate stimulus to the respondents by painting a concrete real-life scenario at work which could evoke different responses. The vignette described the mental health condition of a female co-worker, who was either suffering from a major depression disorder or a general anxiety disorder. Symptoms were outlined, which met the criteria of a general anxiety disorder or major depression disorder and could be observed in the workplace setting prior to the absence of the co-worker due to her mental illness. At the end of the vignette, the female co-worker returns to work after her eight-week sickness leave and discloses her mental illness to a colleague (see Appendix A). The decision fell on these two disorders, as they show the highest prevalence in the working population as well as lead to long periods of illness with subsequent reintegration to employment (2, 64). The portrayal of a female co-worker in the vignette was designed to control stigmatization due to gender and is in accordance with data indicating an overrepresentation of the female population regarding the elected mental health conditions (1).

#### Affects subscale

As there is a paucity of measures assessing emotional responses within mental illness stigma research, we conducted a comprehensive literature search in order to depict the affective component of our scale as thoroughly and broadly as possible. As a result, 39 items were originally framed for the affective subscale, encapsulating positive and negative prejudicial attitudes. The items originated mainly from the circumplex model of affect (65) as well as the Positive and Negative Affect Schedule (66) and were additionally complemented by affective adverbs deemed relevant to the workplace setting by an expert group consisting of three other researchers in the field of IO psychology. The choice fell on these two questionnaires since they map prototypical emotions and affective states and are both reliable and validated measures as well as have been widely employed. The circumplex model (65) pictures the structure of affective experience on two bipolar axes, one ranging from pleasure to unpleasure, characterized as valency, and the other from activation to deactivation, defined as arousal, which together describe each elementary affective condition. Emotions derived from the circumplex model of affect included, *inter alia*, anger, irritability, tension, fatigue, and calmness. The PANAS (66) encompasses a two-factorial structure resulting in positive and negative affectivity. Further items taken from the PANAS scale delineated hostility, determination, fear, and excitement. Supplementary items considered to be important to the study of stigmatizing attitudes toward individuals with mental illness in the workplace were discussed and assessed within the expert panel and finally added to the subscale. All affective items chosen were selected to be uniformly distributed around the dimensions of valency and arousal, referring to the circumplex model. German versions of the questionnaires were used (67). Following the vignette, the respondents were presented with the open sentence “*In such a situation, I feel… (e.g., disturbed)*.” Afterwards they were asked to gage the likelihood of each emotion they experiencing in a situation as described in the vignette on a five-point Likert scale ranging from 1 (*very unlikely*) to 5 (*very likely*). To obtain a spontaneous and intuitive response from the participants, we decided to present the emotional items as adverbs and not as nouns.

#### Cognitions subscale

Since attitudinal measurements of occupational aspects regarding mentally ill employees are sparse, the cognitive dimension of attitude was drawn primarily from five different questionnaires and studies assessing work-related attitudes toward various employment groups (e.g., toward older workers) resulting in 27 items, forming the cognitive component of our scale. Via an expert panel suitable items from each questionnaire used were thoroughly discussed and selected for our cognitive sub-scale. Other items were excluded due to their redundancy. If necessary, items were translated into German via back-translation technique and rephrased to relate to our specific return-to-work context of mentally ill employees. Correspondingly, 14 items from the “Attitudes toward older workers” scale by Kluge and Krings (68) were chosen, further six items were derived from a survey by Popovich et al. (60), which assessed attitudes toward individuals with physical and mental disabilities in the workplace and four items were drawn from a list of character traits and job skills regarding female and male applicants from a study by Steffens and Mehl (69). Cognitive statements extracted from these questionnaires and surveys determine opinions and beliefs toward specific employment populations about their performance abilities, adaptive capacities as well as social skills in the working environment which we regarded equally important when assessing work-related attitudes toward individuals with mental illness in the return-to-work process. Finally, two items originated from mental health surveys about general attitudes toward mentally ill people, which were transferred to the professional context of our scale (48, 70). The final sub-scale records work-related cognitions toward mentally ill co-workers concerning their professional and social competence as well as performance. Following the emotions sub-scale, participants were presented with the open sentence “*Mrs M. … (e.g., needs more assistance than other colleagues)*” and the subsequent 27 cognitive statements referring to the mentally ill co-worker described in the vignette. Respondents were asked to rate the likelihood of each cognition that might occur to themselves in a situation depicted in the vignette on a five-point Likert-scale ranging from 1 (*very unlikely*) to 5 (*very likely*).

#### Behaviors subscale

The behavioral subscale follows the train of thought of social distance by Bogardus (71). The social distance approach assesses a respondent’s readiness to engage in various types of relationships with a target person with items differing in the closeness of the association a respondent is asked to self-disclose. This concept has been a staple in social science research and has been adapted to different frameworks as well as target groups. Consequently, five items referred to the social distance conceptualization and were modified to fit the context of vocational reintegration. In addition, seven items were obtained from the aforementioned survey by Popovich et al. (60) as well as further two items from the questionnaire by Kluge and Krings (68). If necessary, items were back-translated and adjusted to suit our scale. Furthermore, three items were newly formulated to amplify the assessment of prosocial and approach behavior to attain a more complete picture of the behavioral possibilities. Thus, the behaviors subscale comprised originally 17 items tapping into various social interactions as well as prosocial intentions within the workplace environment. Following the cognitions subscale, participants were presented with the open sentence “*I would … (e.g., take a job where I would have to work closely with her)*” and the subsequent 17 behavioral options the respondents could choose to show or decline toward the mentally ill co-worker described in the vignette on a five-point Likert-scale ranging from 1 (*very unlikely*) to 5 (*very likely*).

#### Procedure

The study received ethical approval of the ethics committee of the authors’ university. The questionnaire was developed as an online tool via SoSci Survey to reach as many people as possible (72). Participants were recruited by a mailing list of a German university and through the snowball technique. First participants read a short cover story stating that the study was about improving working conditions to get as many unbiased and authentic answers as possible. After respondents had given their informed consent to participate and had read the technical explanation of the procedure, they completed the questionnaire. Afterwards, a full disclosure of the true research interest of the questionnaire was provided.

### Other measures and their hypothesized relationships with the new scale

To evaluate the construct validity of a new measure, ideally its relationship with an already exiting measure of the construct should be determined. In the absence of the latter, we explored the association between our new scale and a general stigma scale regarding mental illness to investigate the convergent validity of the new instrument. As to discriminant validity, it was expected that the new scale would not be correlated with socially desirable responding, a construct presumed to be unrelated to stigmatizing attitudes toward mental illnesses (57). Since experience with mental illness as well as self-esteem have been consistently linked to stigma related to mental illness, we analyzed the relationship between the new measure and exposure to mental illness and the respondent’s reported self-esteem indicating its criterion/concurrent validity (40, 51, 73). The measures are outlined below.

### Measures

#### Community attitudes to mental illness inventory

Taylor and Dear (43) employed the Opinions about Mental Illness as a conceptual basis for the development of the CAMI scale and reported satisfactory internal reliability for their measure. The instrument includes 40 five-point Likert scaled items, resulting in the four sub-scales authoritarianism, benevolence, social restrictiveness, and community mental health ideology (e.g., “One of the main causes of mental illness is a lack of self-discipline and will-power”). Its major strength lies in its assessment of a broad range of generic attitudes toward mentally ill people as well as the exploration of opinions about mental health treatment facilities. The current study utilized the German version of the scale, which showed close correspondence between the German version and the original inventory with regards to socio-demographic measures as well as factor dimensionality (44). A higher score on this scale represents a greater level of negative attitudes toward mentally ill people. It was anticipated that there would be a positive correlation between the new measure and the CAMI. A significant relationship between the CAMI and the new scale would equally provide support for the convergent validity of our new scale. In the present sample alpha reliability was high, with a Cronbach’s alpha value of 0.92.

#### Balanced inventory of desirable responding

Social desirability bias has been shown to be operative in the assessment of stigma and of less acceptable as well as less normative societal attitudes, which in turn may restrict potential findings (50). Thus, it should be examined whether less stigmatization toward the mentally ill is a result of authentic positive attitudes toward mentally ill people or whether it is merely an effect of socially desirable responding. To that end the 3-item 7-point Likert scaled Impression Management subscale of the original Balanced Inventory of Desirable Responding (74) in German (75) was administered to the participants of the current study (e.g., “I received too much change from a salesperson without telling him or her”). Winkler and his colleagues reported acceptable internal reliability as well as external validity for the subscales of the short version (75). It was hypothesized that there would be no significant associations between the new measure and the BIDR-Impression Management subscale since social desirability is assumed to be unrelated to attitudes toward mental illnesses (57). Streiner (76) argued that inter-item correlations should be considered, especially for short scales, because alpha depends on the length of the scale and the breadth of the measure. Clark and Watson (77) suggest that for scales measuring broad traits, an average inter-item correlation of at least 0.15 should be achieved. Note that the mean inter-item correlation was 0.27 for impression management in our sample.

#### Revised self-esteem scale

This 10-item German measure, using a 4-point Likert scale, was partially revised by Von Collani and Herzberg (78) and is based on Rosenberg’s Self-Esteem Scale (79). It determines a person’s global evaluation of his or her worthiness as a human being [e.g., “On the whole, I am satisfied with myself”] and shows satisfactory internal reliability. The theoretical reasoning underlying the selection of self-esteem as an indicator for the criterion validity of the new scale are the six levels of origins resulting in stigmatization by Haghighat (2001) (80). Accordingly, on a psychological level the process of stigmatization is based on social comparisons, which constitute our concept of self and others. By degrading others, especially minorities and seemingly societal deviators, those with low self-esteem can boost their own self-confidence and well-being. Hence, the presence of a stigmatized person or someone who is less fortunate provides psychological gains for the stigmatizer. Indeed, low self-esteem has been repeatedly found to be associated with negative evaluations of people with disabilities (81, 82). Thus, an inverse association between self-esteem and negative attitudes toward individuals with mental illness was expected. Respondents with higher scores on the self-esteem scale will tend to hold fewer negative attitudes toward people with mental disorders described in the vignette. In the present sample alpha reliability was high, with a Cronbach’s alpha value of 0.89.

#### Contact/experience with mental illnesses

Previous experience or exposure to mental illnesses (either due to own mental disorder or contact with mental illness due to personal, professional, or general circumstances) was measured by a single yes/no/not specified item. Intervention research has illustrated that contact and personal experience with mental disorders helps mitigating stigmatizing processes (83, 84). Therefore, it was hypothesized that there would be a negative correlation between past exposure to mental illness and stigmatizing attitudes toward individuals with a mental disorder described in the vignette.

#### Demographic characteristics

After completing the previous instruments, self-reported gender, age, years of education and current work situation were recorded. To control for acquiescence, participants were asked which topic they thought the study was actually about with a single item in an open answer format. For each demographic variable, respondents had the option of refusing to provide information by choosing “unstated.”

### Results

#### Item selection and factor structure

Firstly, item discrimination indices were calculated to determine the degree of differentiation of each item within the group of respondents. Following conventional recommendations, we only retained items with discrimination indices between 0.4 and 0.7 (85). In a second step, we performed a Principal Component Analysis (PCA) with the remaining 48 items to assess the pattern of factor loadings and variance explained. The oblique rotation by means of the Promax method was employed on the premise that the factors are assumed to correlate and as Promax rotation is the method of choice for oblique proceedings (86). Bartlett’s Test of Sphericity [*χ*^2^ = 3235.74, (*df* = 1,128), *p* < 0.001] and a Kaiser-Meyer-Olkin value of 0.86 confirmed the suitability of examining the factor structure of the data. A cutoff value of 0.5 was applied to meet strict standards for adequate factor loadings for each of the items as well as content-related considerations regarding comprehensibility and redundancy of the items (87, 88). As we wanted to ensure that only parsimonious and functional items were ultimately included, each final subscale consisted of a maximum of 10 items. Thus, factor loadings with the smallest loadings on the corresponding dimension were eliminated. Following this two-step analysis, the final questionnaire was shortened to 22 items, resulting in 7 items for the affective component, 10 items for the cognitions dimension and 5 items for the behavioral component.

In consonance with our theoretical attitudinal framework, the PCA yielded three distinct factors with an eigenvalue greater than 1, explaining 57.9% of the total variance. As can be seen in Table 1, all of the items of the emotion’s subscale can be located in the region of unpleasant deactivation to unpleasant activation, illustrating negative affectivity toward the mentally ill person described in the vignette. The 10 items of the cognitive factor embody a wide range of evaluations of professional skills and competences and the 5 items of the behavioral component reflect distancing or approaching behavioral possibilities. All items loaded exclusively on only one of the three factors, except for one item of the behavioral subscale which also loaded on the cognitive dimension. Due to theoretical conceptualizations, we still allocated this item to the behavioral subscale (see Table 1). As expected, all three subscales correlated significantly with each other, indicating one superordinate factor, that is stigmatizing attitude. The strongest association was found between cognitions and behavior [*r* (102) = 0.63, *p* < 0.001], followed by a moderate correlation between cognitions and emotions [*r* (102) = 0.34, *p* < 0.001] and a small association between behavior and emotions [*r* (102) = 0.21, *p* < 0.05]. Consequently, the three factors appear to share a similar foundation, yet each representing different dimensions of the overarching component stigmatizing attitude. The 22-item scale was found to be highly internally consistent, with a Cronbach’s alpha value of 0.91. Equally, although varying in their number of items, the three subscales showed high internal consistency: The Cronbach’s alphas for the Emotions subscale, the Cognitions subscale and the Behavioral subscale were 0.89, 0.90 and 0.83, respectively. Descriptive statistics for each subscale and their correlations with the overall stigma scale are presented in Table 2.

**Table 1:** Factor loadings and communalities for final three-factor solution in Study 1.

Item	Affects	Cognitions	Behaviors	Communality
Nervous	0.86	−0.05	−0.01	0.71
Stressed	0.90	−0.06	−0.07	0.77
Scared	0.75	0.09	−0.05	0.60
Exhausted	0.77	−0.05	0.06	0.59
Ashamed	0.64	−0.05	0.14	0.44
Confused	0.79	0.03	−0.06	0.63
Disturbed	0.67	0.11	0.10	0.55
Ms. M. can be just as successful professionally as others. (−)	0.01	0.66	0.07	0.50
Ms. M. is not able to make important decisions.	0.06	0.79	−0.19	0.53
Ms. M. needs more assistance than other colleagues.	0.01	0.56	0.10	0.38
Ms. M. slows down the speed at which work is completed.	−0.04	0.76	0.08	0.64
Ms. M. is more difficult to train for new work tasks than other colleagues.	0.10	0.82	−0.15	0.61
Ms. M. is more prone to physical impairments (e.g., back pain, headaches, etc.).	0.26	0.54	0.06	0.50
Ms. M. is a capable employee. (−)	−0.14	0.69	0.15	0.55
Ms. M. will work just as hard as anyone else. (−)	−0.07	0.69	0.06	0.49
Ms. M. has certainly decreased in her performance.	−0.04	0.86	−0.21	0.57
Ms. M. cannot keep up with the pace of work at work.	−0.01	0.76	−0.01	0.57
I would share my office or desk with her. (−)	−0.04	−0.04	0.84	0.67
I would take a job where I would have to work closely with her. (−)	0.04	0.01	0.76	0.60
I would put her forward for promotion. (−)	0.01	0.38	0.51	0.62
I would carpool with her using my car. (−)	0.09	−0.16	0.88	0.67
I would designate her as my proxy in my absence. (−)	−0.07	0.45	0.44	0.58

*Note. N = 104. (−) Reverse coded*

**Table 2:** Descriptive statistics, reliabilities (on the diagonals), and correlations for study 1 (*N* = 104).

Variable	*M*	*SD*	1	2	3	4	5	6	7
1. WMISS	2.59	0.62	0.91						
2. Affects Subscale	2.09	0.83	0.68**	0.89					
3. Cognitions Subscale	2.77	0.76	0.89**	0.34**	0.90				
4. Behaviors Subscale	2.95	0.78	0.73**	0.21*	0.63**	0.83			
5. CAMI	1.88	0.49	0.47**	0.11	0.50**	0.51**	0.92		
6. BIDR-IM	4.55	1.21	−0.06	−0.07	0.02	−0.15	−0.11	0.27	
7. SES-R	3.00	0.55	−0.20*	−0.22*	−0.16	−0.07	−0.08	0.02	0.89

WMISS, Workplace Mental Illness Stigma Scale; CAMI, Community Attitudes Toward the Mentally Ill; BIDR-IM, Balanced Inventory of Desirable Responding-Impression Management Subscale; SES-R, Self-Esteem Scale Revised.

**p < 0.01, two-tailed tests. *p < 0.05, two-tailed tests.

#### Convergent and discriminant validity

As hypothesized, results revealed that the new scale was significantly correlated with the CAMI [*r* (102) = 0.47, *p* < 0.001]. Since the CAMI inventory assesses global evaluations about mental illnesses and the new scale measures specific attitudes toward mental illnesses within the return-to-work process, the association between the two scales is expectedly not particularly high, indicating that the new instrument determines disparate attitudes of those gaged in the CAMI. Significant associations emerged from the correlations between the cognitive subscale and the CAMI scale, [*r* (102) = 0.50, *p* < 0.001], as well as between the behavioral subscale and the CAMI measure [*r* (102) = 0.51, *p* < 0.001]. The affective subscale and the CAMI scale showed no significant correlation, since the CAMI focuses on cognitive and behavioral tendencies toward mentally ill people rather than emotional responses [*r* (102) = 0.11, *p* = 0.266]. As for discriminant validity, no significant correlation was found between the new scale and the social desirability measure BIDR-Impression Management [*r* (102) = −0.06, *p* = 0.565] as well as between each of the subscales and the BIDR-Impression Management, equally supporting our assumptions (see Table 2).

#### Criterion validity

In order to investigate criterion validity of the new measure, we referenced self-esteem as a relevant external criterion. As predicted, self-esteem was found to correlate significantly and inversely with the new scale [*r* (102) = −0.203, *p* < 0.05]. Hence, the higher the respondent’s self-esteem, the more positive his or her attitudes were toward the person with a mental illness. As for the subscales, only the correlation between the affective subscale and the self-esteem scale reached significance [*r* (102) = −0.22, *p* < 0.05]. This can be due to the circumstance that self-esteem taps into self-evaluations about the feelings concerning one’s global self-worth (89).

#### Associations between exposure/experience with mental illness and the new scale

Contrary to our assumptions, there were no significant differences between exposure/contact to mental illness (yes/no) and the overall stigma score [*t* (100) = 1.47, *p* = 0.144]. Equally, a one-way MANOVA found no statistically significant differences between exposure/contact to mental illness on the combined subscales, *F* (3, 98) = 1.63, *p* = 0.187, partial η^2^ = 0.05, Wilk’s Λ = 0.95. However, analyzes on a descriptive level show that the mean values indicate a tendency toward a negative association.

#### Associations between the new scale and sociodemographic variables

We examined the relationship between basic demographic characteristics and our new scale by computing Pearson and Spearman correlations for the continuous and ordinal variables (age and level of education) and t-tests analyzes for the dichotomous variable (gender). No significant association was found between age and the new scale, neither for the overall stigma scale [*r* (102) = −0.033, *p* = 0.742], nor for the three subscales. The analyzes between level of education and the new measure also rendered no significant correlations, neither for the overall stigma score, Spearman’s ρ = −0.106, *p* = 0.284, nor for the three subscales. As to gender, no significant differences were found between women and men on the overall stigmatizing scale, [*t* (102) = −0.662, *p* = 0.403]. However, subscale analyzes elicited gender differences on the emotional subscale. Post-hoc univariate ANOVAs were conducted for every dependent variable after a one-way MANOVA showed a statistically significant difference between the gender on the combined dependent variables, *F* (3, 100) = 4.87, *p* < 0.05, partial η^2^ = 0.13. Results demonstrated a statistically significant difference between the scores of the female and male participants for the affective subscale, *F* (1, 102) = 7.23, *p* < 0.05, partial η^2^ = 0.07, indicating that female participants responded higher in negative affectivity toward the mentally ill person in the vignette than male participants, but not for the other two subscales, cognitions, *F* (1, 102) = 0.01, *p* = 0.924, partial η^2^ = 0.00 and behavior *F* (1, 102) = 3.29, *p* = 0.073, partial η^2^ = 0.03.

### Discussion

In this first study, we developed a multidimensional mental illness stigma questionnaire in the workplace based on the emotional-cognitive-behavioral framework within the attitudinal research. By means of item- and reliability analyzes as well as PCA the original item pool of 83 items could be reduced to a parsimonious final set of 22 items. Equally the originally proposed three factor model could be demonstrated via exploratory factor analysis. Small to moderate intercorrelations between the subscales underpin the notion of a superordinate factor stigmatization. As for convergent validity, bivariate correlations with the CAMI measure further support our new questionnaire and show its incremental validity beyond the established inventory, since it is specifically tailored to capture attitudes within the workplace regarding mentally ill co-workers on the emotional, cognitive, and behavioral level. In support of our assumptions concerning discriminant validity, there was no significant correlation between our new measure and its subscales and the social desirability scale. Regarding the criterion validity of our new scale, results show a significant inverse correlation between self-esteem and the overall stigma scale as well as the affective subscale.

Contrary to our assumptions, there was no significant association between exposure to mental illness and the stigma scale. This could be partly due to the fact that contact/exposure to mental illness was only measured with a single item without assessing the quality of the experience or exposure. As for sociodemographic variables, significant differences between male and female participants were only found on subscale level, indicating that female participants showed more negative affectivity toward the mentally ill co-worker described in the vignette than their male counterparts.

## Study 2: replicating construct validity and criterion validity

In Study 2, we aimed to examine the consistency of the factors identified in the previous study by collecting a new sample and conducting Confirmatory Factor Analysis (CFA) to evaluate the fit of our new measure. Additionally, in order to replicate and extend convergent and criterion validity, we examined the relations of the new stigma questionnaire with other relevant measures and personality traits.

### Methods

#### Participants

The participants were collected using an online questionnaire on the platform Sosci Survey and by a mailing list of students and staff from a university in Germany. In total, 147 participants took part in this study (81% were female, others were male), with an age distribution of 58.5% between 18 and 29 years old, 22.4% between 30 and 39 years old, 8.2% between 40 and 49 years old, 6.1% between 50 and 59 years old, and 4.8% between 60–69 years old. 42.2% had completed a university degree, 52.4% were employed. Overall, 79.6% reported having had experiences with mental illness, 42.9% described this contact or experience as partly positive and partly negative.

#### Procedure

First participants read a short cover story stating that the study was about improving working conditions to get as many unbiased and authentic answers as possible. After respondents had given their informed consent to participate and had read the technical explanation of the procedure, they completed the questionnaire. At the end of the study, the participants were fully debriefed and thanked.

### Measures

#### Workplace mental illness stigma scale

Based on the results of the previous study, the WMISS contained the revised final questionnaire of 22 items, 7 items in the affective subscale, 10 items in the cognitive subscale and finally 5 items in the behavioral subscale.

#### Mental illness microaggressions scale

After assessing convergent validity with the CAMI in the previous study, we were interested in examining the association between our new scale and microaggression behaviors perpetrated toward persons with mental illness. Microaggressions are classified as subtler forms of discrimination which tend to be implicit prejudicial manners of communication. As there is no German scale available, we translated the MIMS-P (57) into German using the forward-backward procedure (e.g., “If someone I’m close to told me that they had a mental illness diagnosis, I would be careful in case they ‘snap’”). The MIMS-P contains 17 items, shows good internal consistency, and encompasses microaggression behaviors by three subscales (Assumption of Inferiority, Patronization, Fear of Mental Illness). A positive correlation between the new stigma scale and the MIMS-P was expected. In our current study alpha reliability was satisfying, with a Cronbach’s alpha value of 0.86.

#### Need for cognitive closure (German Short Scale)

The need for cognitive closure (NFC) is defined as a personality trait which affects cognitive processing, with individuals high in NFC tending to eschew uncertainty. The desire to reduce confusing and ambiguous information usually results in effort minimizing cognitive strategies, i.e., employing quickly accessible and stereotypical information in judgment formation (90). Indeed, NFC has been linked to a large scope of racial and gender prejudices (91). The inclination to utilize the most readily available information even hinders the likelihood of perspective taking when reading about a person with whom one is not identified with, as one’s own viewpoint is likely to be the most rapidly present (92). Accordingly, we proposed a positive relationship between the need for cognitive closure and stigmatization on the affective, cognitive, and behavioral level. The 16-items German Short Scale for Need for Cognitive Closure (e.g., “I do not like it when a person’s statement is ambiguous.”) with an acceptable internal consistency was used in the Study 2 (93). In our current sample alpha reliability was satisfying, with a Cronbach’s alpha value of 0.85.

#### Social Dominance Orientation (German Short Scale)

Social dominance orientation (SDO) refers to a belief system with a preference for inequality among social groups and hence a favoring of a hierarchical society (94). This tendency has been associated with biased attitudes against a range of stigmatized targets, including minorities (95) and higher-weight individuals (96). Consequently, a positive relationship between social dominance orientation and the WMISS was expected. Social dominance orientation was assessed by the 3-items German short scale KSDO-3 (e.g., “Every society needs groups that are at the top and others that are at the bottom.”) which shows acceptable reliability (97). In the present sample alpha reliability was acceptable, with a Cronbach’s alpha value of 0.61.

#### Balanced inventory of desirable responding

With the intention of replicating and corroborating our findings of Study 1, socially desirable responding behavior was measured with the same Impression Management subscale of the BIDR as in Study 1. No significant correlation was hypothesized for the relationship between socially desirable responding and the WMISS. The mean inter-item correlation of the impression management subscale was 0.22 in our second study.

#### Demographic characteristics

Participants’ information about their age, gender, years of education as well as professional background were collected.

#### Contact/experience with mental illnesses and quality of contact

Previous experience or exposure to mental illnesses (either due to own mental disorder or contact with mental illness due to personal, professional, or general circumstances) was measured by a single yes/no/not specified item. Equally a single item for the assessment of quality of contact was used, participants should classify their experienced contact as positive, negative, or partly positive/partly negative.

### Results

Several fit indices were examined, including standardized root mean square residual (SRMR), the standard fit index (NFI), the comparative fit index (CFI) and the root-mean-square error of approximation (RMSEA). Values of 0.90 or higher are indicators of good fit for the NFI and CFI; values between 0.05 to 0.09 are generally thought to be reasonable for the RMSEA; while values lower than 0.05 indicate an acceptable fit for the SRMR (98).

The indices of the original model did not reach the benchmarks for acceptable fit: *χ*^2^ (206) = 432.92, *p* < 0.001, SRMR = 0.11, NFI = 0.75, CFI = 0.85, RMSEA = 0.08. After careful consideration and theoretical reasoning, we referred to the modification indices and added five pairs of error covariances (KG01, KG02, KG03, DI18, DI20, DI21, DI22), as they were reversely coded and within the same subscale. The overall fit improved: *χ*^2^ (201) = 334.35, *p* < 0.001, SRMR = 0.11, NFI = 0.81, CFI = 0.91, RMSEA =0.07 (see Table 3).

**Table 3:** Fit indices for the workplace mental illness stigma scale in study 2.

	*χ* ^2^	*df*	CFI	TLI	RMSEA	RMSEA CI	SRMR	Δ*χ*^2^	Δ*df*	*p*
Model A’										
Modified Three-factor model	334.35	201	0.91	0.90	0.07	[0.05, 0.08]	0.09	―	―	―
Model A										
Three-factor model	432.92	206	0.85	0.83	0.09	[0.08, 0.09]	0.09	98.57	5	<0.01
Model B										
Single-factor model	1022.53	209	0.46	0.40	0.16	[0.15, 0.17]	0.18	688.18	8	<0.01

*N* = 147. Model A and Model B are compared to Model A’ (proposed model).

Additionally, the 3-factor subscale structure with a superordinate factor proved to be superior in fit to a model that estimated only a single undifferentiated stigma factor: Δ*χ*^2^ (8, *N* = 147) = 688.19, *p* < 0.001, SRMR = 0.22, NFI = 0.41, CFI = 0.46, RMSEA = 0.16 (see Table 3). Moreover, all items loaded significantly and moderately to strongly on their respective factors.

Similar to Study 1, the 22-items scale was found to be highly internally consistent, with a Cronbach’s alpha value of 0.89. Equally, the three subscales demonstrated high internal consistency: The Cronbach’s alphas for the Emotions subscale, the Cognitions subscale and the Behavioral subscale were 0.91, 0.84 and 0.84, respectively (see Table 4).

**Table 4:** Descriptive statistics, reliabilities (on the diagonals), and correlations for study 2 (*N* = 147).

Variable	*M*	SD	1	2	3	4	5	6	7	8
1. WMISS	2.77	0.63	0.89							
2. Affects Subscale	2.54	1.03	0.73**	0.91						
3. Cognitions Subscale	2.91	0.72	0.84**	0.32**	0.84					
4. Behaviors Subscale	2.81	0.82	0.64**	0.14	0.52**	0.84				
5. MIMS-P	2.10	0.47	0.56**	0.36**	0.50**	0.42**	0.86			
6. NFC	3.35	0.69	0.33**	0.31**	0.27**	0.11	0.43**	0.85		
7. SDO	1.99	0.79	0.24**	0.03	0.25**	0.34**	0.33**	0.17*	0.61	
8. BIDR-IM	3.62	0.95	−0.09	−0.06	−0.03	−0.13	−0.19*	−0.11	−0.15	0.22

WMISS, Workplace Mental Illness Stigma Scale; MIMS-P, Mental Illness Microaggression Scale-Perpetrator Version; NFC, Need for Cognitive Closure Scale; SDO, Social Dominance Orientation Scale; BIDR-IM, Balanced Inventory of Desirable Responding-Impression Management Subscale.

**p < 0.01, two-tailed tests. *p < 0.05, two-tailed tests.

#### Convergent and discriminant validity

As expected, the new WMISS correlated significantly and strongly with the MIMS-P, [*r* (145) = 0.56, *p* < 0.01]. Correspondingly all subscales showed moderate to strong correlations with the MIMS-P (see Table 4). For discriminant validity the relationship between the WMISS and socially desirable responding behavior was reassessed. As hypothesized, the new scale and its subscales did not show any significant relationship with the BIDR-Impression Management subscale, [*r* (145) = −0.09, *p* = 0.292]. All subscale correlations are shown in Table 4.

#### Criterion validity

The new measure was correlated with a set of personality variables in order to further examine its criterion validity. As expected, the WMISS correlated positively and significantly with the need for cognitive closure, [*r* (145) = 0.33, *p* < 0.01] and social dominance orientation, [*r* (145) = 0.24, *p* < 0.01]. For all subscale correlations see Table 4.

#### Associations between the WMISS and contact with mental illness/ quality of contact

Contrary to our expectations, a one-way ANOVA and a one-way MANOVA, respectively revealed no significant difference between the different groups of experienced contact or exposure to mental illness on the overall stigma scores, *F*(2, 144) = 0.13, *p* = 0.881, nor on the subscale scores, *F*(6, 284) = 0.21, *p* = 0.973. Likewise, quality of contact, as assessed in three categories (positive, negative, and partly positive, partly negative) neither affected significantly the overall stigma scores, *F*(3, 113) = 1.54, *p* = 0.208, nor the subscale scores, *F*(2, 270.30) = 1.07, *p* = 0.387.

#### Associations between the WMISS and sociodemographic variables

Similar to Study 1, no significant correlations were found between level of education and overall stigma scores or subscale scores. Also results showed no significant association between age and overall stigma scores and its subscale scores, except for the behavioral subscale, with a small positive correlation, Spearman’s ρ =0.17, *p* < 0.05. No significant difference emerged from the analysis of female and male participants regarding their overall stigma scores, *t*(145) = −1.53, *p* = 0.129, nor their subscale scores, *F*(3, 143) = 1.92, *p* = 0.129. With regards to occupational status, results showed no significant differences between employed and non-working participants for their overall stigma scores, *t*(142) = 1.13, *p* = 0.262, and for their subscale scores, *F*(3, 140) = 1.09, *p* = 0.358.

### Discussion

Study 2 provides further evidence for the proposed factor structure of the WMISS. The CFA results substantiated Study 1 and supported the three-factor model with stigma being the superordinate factor in comparison to an undifferentiated one-factor model.

Additionally, Study 2 yielded further support for the new measure’s convergent and discriminant validity. The WMISS and its subscales correlated significantly and moderately to strongly with the MIMS-P. As in Study 1, the new measure and its subscales did not show any significant relationship with the BIDR-Impression Management subscale, corroborating its discriminant validity. Besides, findings in Study 2 were in line with the new scale’s suggested association with additional relevant personality variables, indicating criterion validity. The WMISS correlated moderately and significantly with the need for cognitive closure and social dominance orientation.

As to contact with mental illness and quality of contact with mental illness, analysis showed no significant difference in the tendency of stigmatization. This is surprising, since contact theory states that positive contact leads to tolerance and acceptance between groups (99). Nonetheless it could be that level of contact and intimacy is equally as important, for it makes a difference whether one comes into contact with mental illness by a relative or by a stranger. No age and education differences were found, replicating findings from the previous study. Similarly, analyzes rendered no differences between the occupational status and stigmatization. However, also no gender differences were found on overall and subscale level, which is contradictory to Study 1. Yet, this goes to show that gender differences concerning stigmatization need to be researched further, since findings are heterogenous and incoherent.

Taken together, our second study provided support for the proposed three-factor model as well as for the convergent and discriminant validity of the newly developed measure by looking at their correlations with conceptually similar as well as theoretically unrelated concepts. Still further research is needed to explore the new questionnaire’s predictive and incremental validity.

## General discussion

In our present research we sought to devise a new multidimensional workplace stigmatization measure with sound theoretical foundations and reasonable psychometric properties. Derived from the affective-cognitive-behavioral framework of attitude (39), we created, *a priori*, an instrument consisting of three dimensions, representing the overall common core of stigmatizing attitude. After item selection, the initial item pool of 83 items was reduced to a parsimonious set of 22 items. Statistical analyzes yielded small to moderate and strong correlations between the subscales, revealing, on the one hand, disparate and separate components, and on the other hand, illustrating a mutual overarching factor. In both studies the new WMISS proved to be highly internally consistent, and its proposed three-factor structure was equally supported across the two studies. More precisely, the three-factor model with a superordinate construct showed a significantly better fit to the data than alternative models with an undifferentiated factor. Convergent and discriminant validity were demonstrated by moderate and high correlations (e.g., CAMI) or zero correlations (BIDR) with pertinent measures. Furthermore, construct validity of the new scale was supported by significant positive associations with two personality characteristics, namely need for cognitive closure and social dominance orientation, which is also in line with previous research, linking these personality traits to biased and discriminatory behavior (95). Moreover, inverse correlations between self-esteem and the WMISS were found for the overall stigma score as well as for the affective subscale, supporting the notion, that individuals with a lower self-esteem tend to hold more stigmatizing attitudes toward seemingly deviant people than individuals with a higher self-esteem (81, 82).

Surprisingly, neither self-reported exposure to mental illness nor the quality of this exposure or experience seemed to impact stigmatization. However, several studies support the social contact hypothesis in reducing stigma against individuals with mental health issues by facilitating inter-group relations [e.g., (100, 101)]. In our study contact and quality of contact was measured by using a single “yes/no” or “negative/positive/partly positive, partly negative” item. It could be that exposure to mental illness requires a more detailed assessment of its form of social contact and experience, since it makes all the difference whether one experienced mental illness himself/herself, through a relative or colleague. The level of contact report which lists 12 situations in which intimacy of contact with mental illness is varied could be a useful tool to expand knowledge in that regard and should be considered for future research (102).

### Theoretical and practical implications

To our knowledge, this is the first multidimensional mental illness stigma scale which centers around the occupational setting and the return of mentally ill employers to their workplace. The newly developed WMISS not only closes a remarkable gap in research on stigmatizing attitudes toward the mentally ill but also offers promising prospects for workplace reintegration programs. Thus far, instruments on stigmatizing attitudes have neglected the multidimensional aspect of attitudinal conceptualizations and have mostly focused on the cognitive and behavioral part of stigmatization, while overlooking the affective component of stigmatizing attitudes (50). However, the consideration of a multidimensional approach is especially crucial when studying attitudes toward mentally ill people since any measure will inevitably not reflect a comprehensive picture of attitudes when assessing only one or two dimensions of the attitudinal framework (39). As pointed out by Findler et al. (63) with regards to people with disabilities, this might even lead to false conclusions being drawn, when, for instance, only concentrating on discriminating behavioral actions, which are generally less likely to be admitted, while at the same time disregarding negative affective responses concerning mentally ill people and thus depicting a more favorable attitude toward them. Statistical support for the multidimensional perspective is also demonstrated, when evaluating the correlational analyzes between the CAMI and the subscales of the WMISS: The CAMI questionnaire captures mostly cognitive and behavioral elements of stigmatizing attitudes perpetrated against mentally ill individuals, hence only the cognitive and behavioral subscales of our new scale were found to be strongly associated with the CAMI, while the affective subscale showed no significant correlation with the latter. Besides most mental illness stigma measures were framed within a more general context of stigmatization, which could potentially result in less valuable implications when its outcomes are to be applied to a specific setting. This is especially important when it comes to outlining intervention strategies. A questionnaire which assesses stigmatizing attitudes within a specific life area can provide fruitful insights not only into understanding marginalization and discrimination regarding a distinct context but might also function as a helpful tool for targeted and effective intervention programs and their subsequent evaluation. For example, mental disorder de-stigmatization campaigns within the occupational sector require a different modus operandi than broad-based anti-discrimination initiatives, as employees with mental health issues face particular workplace barriers which oftentimes revolve around their abilities, stamina and social skills (10). Specifically, the WMISS could be a beneficial instrument in accompanying and evaluating occupational re-integration programs within the HR sector of companies in order to support job accommodations for people with mental illness, but also as an initial assessment tool for capturing attitudes of colleagues regarding mental illnesses and analyzing mental illness literacy within an organization. Furthermore, most research to date in the field of mental illness stigma has concentrated on severe mental disorders, e.g., schizophrenia, substance abuse disorders [e.g., (11, 12, 83)]. Yet, comparatively little attention has been paid to stigma surrounding depression and generalized anxiety disorder. This is especially alarming given that both depression and generalized anxiety disorder are highly prevalent in the public, crippling in their consequences for those affected and cause long periods of illness and lengthy sickness-related absences from work (64). Our instrument spotlights these two disorders and helps to identify how employees with a depression disorder or generalized anxiety disorder are perceived and treated by their co-workers. This is a crucial step in improving the quality of work experiences of mentally ill employees and refining interventions to create a more supportive and inclusive working environment.

### Strengths, limitations, and future research

The findings of the two studies in this paper provide evidence for the new scale’s factor structure, internal consistency, convergent and discriminant validity as well as criterion validity. One of the major strengths of the present research is the theory-driven adoption of the multi-dimensional perspective of stigmatizing attitudes and its implementation by constructing a vast and extensive initial item pool of 83 items. In this context, particular consideration was given to the comprehensive composition of the affective dimension of the new scale, as stigma-related feelings have long been a missing component in the assessment of stigma-associated processes (50). Yet another strong point of our research lies in the new instrument’s validation by means of two different samples, which increases generalization of the results.

However, still further research is needed to establish the new scale as a sound measure for the assessment of mental illness stigma within the occupational setting. One of the primary limitations of the current study is the relatively small sample size and its homogenous composition. Notably the sample comprised more women than men, higher educated participants than lower educated respondents and more younger participants than middle-aged or older respondents. In addition, in both studies over 70% of the participants reported having had exposure to mental illness. Besides, we only validated the German set of items of the WMISS. Future research should therefore further investigate the new scale’s psychometric properties in different populations and in international samples.

Another potential shortcoming of our studies might be that both of our samples were collected via online tools which can unavoidably lead to self-selection processes, so that only participants with certain personality traits or those who have access to online panels would take part in the studies. Hence, in future studies various formats of recruitment should be considered to expand the representativeness of the research.

Finally, our instrument solely depends on the respondent’s self-reports, resulting in a potential underestimation of the individual’s level of stigmatizing attitudes. In addition, it is important to note that vignette-based studies only depict hypothetical and abstract real-life situations, neglecting non-verbal cues and unconscious information processing through observable signals. Future research could address this drawback by combing non-vignette experimental studies with self-report measures. Thus, the additional assessment of implicit measures, such as behavioral or physiological outcomes in real-life scenarios, will shed further light on the incremental and predictive validity of the WMISS. In fact, previous research has illustrated the significant association between explicit attitudinal evaluations and physical proximity with regards to mental health stigma (103).

A possible extension of our research may also lie in the development of vignettes for other mental illnesses as well as in the depiction of male employees with a mental health status to expand the scope of the WMISS for the workplace setting and to examine labeling effects between the different mental disorders and genders. Additional value for the new inventory could certainly also be generated through the comparison of perceptions and experiences of mentally ill employees concerning workplace stigmatization with our new scale as well as their potential tendencies of self-stigmatization within the occupational context.

## Conclusion

Our newly developed multidimensional WMISS allows researchers and professionals to assess stigma associated with mental disorders within the working environment reliably and thoroughly based on the profound theoretical foundation of attitudinal research. The results obtained in the two studies are indicative of the instrument’s factor structure, reliability, and validity. Hence, the WMISS is a promising measure for use in future studies in further exploring mental illness stigma in the occupational sector as well as designing targeted and tailored intervention programs aimed at modifying attitudes regarding mental illnesses.

## Data availability statement

The raw data supporting the conclusions of this article will be made available by the authors, without undue reservation.

## Ethics statement

The studies involving human participants were reviewed and approved by Ethics Committee of the Psychology Department of the University Marburg. The patients/participants provided their written informed consent to participate in this study.

## Author contributions

NM and KO structured the main ideas and developed the research question. NM developed the design of the studies, conducted the surveys, performed the analyzes, and wrote the first draft. All authors read and approved the final manuscript.

## Conflict of interest

The authors declare that the research was conducted in the absence of any commercial or financial relationships that could be construed as a potential conflict of interest.

## Publisher’s note

All claims expressed in this article are solely those of the authors and do not necessarily represent those of their affiliated organizations, or those of the publisher, the editors and the reviewers. Any product that may be evaluated in this article, or claim that may be made by its manufacturer, is not guaranteed or endorsed by the publisher.
