# Supplementary material for: How to measure mental illness stigma at work: development and validation of the workplace mental illness stigma scale
Source: Front Psychiatry. 2023 Jul 12;14:1225838. doi: 10.3389/fpsyt.2023.1225838 (PMC10369081; doi:10.3389/fpsyt.2023.1225838)
Supplement: Supplementary file 1 [file Table_1.docx]

Appendix A

*The Workplace Mental Illness Stigma Scale (WMISS) – Original German items and English translation*

|  | **Original German version** | **English translation** |
| --- | --- | --- |
| **Vignette 1**  **(Depression Disorder)** | Stellen Sie sich bitte folgende Situation am Arbeitsplatz vor: Bevor Frau M. für 8 Wochen krankheitsbedingt am Arbeitsplatz ausfiel, beobachteten Sie, dass sie häufig müde und reizbarer war.  Ihr fiel es schwer, sich auf ihre Aufgaben und Gespräche zu konzentrieren und sie schien kraftlos und schneller erschöpft zu sein.  Darüber hinaus wirkte sie in den Teamsitzungen und bei Arbeitsaufgaben teilnahms- und interesselos.  Ihnen war aufgefallen, dass sich zudem ihr Gewicht verändert hatte.  Frau M. selbst berichtete, dass sie seit einiger Zeit ein erhöhtes Schlafbedürfnis habe und ihr zunehmend der Antrieb fehle.  Nun kehrt Frau M. an ihren Arbeitsplatz zurück und erzählt, dass sie aufgrund einer depressiven Erkrankung ausgefallen war. | Please imagine the following situation at work:  Before Ms. M was absent from work due to illness for 8 weeks, you observed that she was frequently tired and more irritable.  She found it difficult to concentrate on her tasks and conversations, and she seemed to be powerless and exhausted more quickly.  In addition, she seemed apathetic and uninterested in team meetings and work tasks.  You had noticed that her weight had also changed.  Ms. M. herself reported that she had an increased need for sleep for some time and increasingly lacked drive.  Now Ms. M. returns to her workplace and tells that she had been absent due to a depressive disorder. |
| **Vignette 2**  **(Generalized Anxiety Disorder)** | Stellen Sie sich bitte folgende Situation am Arbeitsplatz vor: Bevor Frau M. für 8 Wochen krankheitsbedingt am Arbeitsplatz ausfiel, beobachteten Sie, dass sie häufig nervös war und zitterte.  Ihnen war außerdem aufgefallen, dass sie bei allgemeinen Arbeitsvorgängen unruhig und generell ängstlicher war.  Frau M. selbst berichtete, dass sie abends schlecht einschlafen könne und seit geraumer Zeit unter einer inneren Anspannung leide.  Weiterhin klagte sie über starkes Herzklopfen, verstärktes Schwitzen und Übelkeit.  Darüber hinaus fühle sie sich allgemein „schwach".  Nun kehrt Frau M. an ihren Arbeitsplatz zurück und erzählt, dass sie aufgrund einer Angsterkrankung ausgefallen war. | Please imagine the following situation at work:  Before Ms. M was absent from work due to illness for 8 weeks, you observed that she was frequently nervous and trembling.  You had also noticed that she was restless and generally more anxious during common work procedures.  Ms. M. herself reported that she had difficulty falling asleep in the evenings and had been suffering from inner tension for some time.  Furthermore, she complained of strong heart palpitations, increased sweating, and nausea.  In addition, she felt "weak" in general.  Now Ms. M. returns to her workplace and tells that she had been absent due to an anxiety disorder. |
| **Affects Subscale** | Viele Menschen empfinden in solch einer Situation eine Vielzahl von unterschiedlichen Gefühlen.  Im nächsten Abschnitt finden Sie eine Reihe von Gefühlen, die in solch einer Situation auftreten können.  Bitte schätzen Sie, als Kolleg:in von Frau M., für jede Zeile ein: Wie wahrscheinlich würden Sie das Gefühl in solch einer Situation verspüren?  Gestört  Durcheinander  Verängstigt  Nervös  Beschämt  Gestresst  Erschöpft | Many people experience a variety of different feelings in such a situation.  In the next section, you will find a number of feelings that may occur in such a situation.  For each line, please rate, as a colleague of Ms. M., how likely you would feel the emotion in such a situation:  Disturbed  Confused  Scared  Nervous  Ashamed  Stressed  Exhausted |
| **Cognitions Subscale** | Viele Menschen haben in solch einer Situation eine Vielzahl von unterschiedlichen Gedanken über die betroffene Person im beschriebenen Beispiel.  Im nächsten Abschnitt finden Sie eine Reihe von Gedankengängen, die in solch einer Situation auftreten können.  Bitte schätzen Sie für jede Zeile ein: Wie wahrscheinlich würde der Gedanke in solch einer Situation bei Ihnen auftreten?  Frau M. benötigt mehr Betreuung als andere Kolleg:innen.  Frau M. ist schwieriger für neue Arbeiten auszubilden als andere Kolleg:innen.  Frau M. ist nicht in der Lage, wichtige Entscheidungen zu treffen.  Frau M. ist anfälliger für körperliche Beeinträchtigungen (z.B. Rückenschmerzen, Kopfschmerzen etc.).  Frau M. verlangsamt die Geschwindigkeit, mit der Arbeiten abgeschlossen werden.  Frau M. kann beruflich genauso erfolgreich sein wie andere.  Frau M. wird genauso hart arbeiten wie jede:r andere.  Frau M. ist eine leistungsfähige Arbeitnehmerin.  Frau M. hat in ihrer Leistungsfähigkeit sicherlich abgenommen.  Frau M. kann mit dem Arbeitstempo bei der Arbeit nicht mithalten. | In such a situation, many people have a variety of different thoughts about the person in the example described.  In the next section, you will find a number of thoughts that may occur in such a situation.  For each line, please rate how likely the thought might occur to you in you in such a situation.  Ms. M. needs more assistance than other colleagues.  Ms. M. is more difficult to train for new work tasks than other colleagues.  Mrs. M. is not able to make important decisions.  Ms. M. is more prone to physical impairments (e.g., back pain, headaches, etc.).  Ms. M slows down the speed at which work is completed.  Ms. M. can be just as successful professionally as others.  Ms. M will work just as hard as anyone else.  Ms. M is a capable employee.  Ms. M has certainly decreased in her performance.  Ms. M cannot keep up with the pace of work at work. |
| **Behaviors Subscale** | Viele Menschen verhalten sich in solch einer Situation auf unterschiedliche Art und Weise gegenüber der betroffenen Person im beschriebenen Beispiel.  Im nächsten Abschnitt finden Sie eine Reihe von Verhaltensweisen, die in solch einer Situation auftreten können.  Bitte schätzen Sie in jeder Zeile ein: Wie wahrscheinlich würden Sie die Verhaltensweise in solch einer Situation zeigen?  Ich würde einen Job annehmen, bei dem ich eng mit ihr zusammenarbeiten müsste.  Ich würde mit ihr mein Büro oder meinen Schreibtisch teilen.  Ich würde mit ihr eine Fahrgemeinschaft unter Benutzung meines PKWs bilden.  Ich würde sie für eine Beförderung vorschlagen.  Ich würde sie in meiner Abwesenheit als meine Stellvertretung bestimmen. | In such a situation, many people behave in different ways towards the person in the example described.  In the next section you will find a number of behaviors that may occur in such a situation.  For each line, please rate how likely you would show the behavior in such a situation.  I would take a job where I would have to work closely with her.  I would share my office or desk with her.  I would carpool with her using my car.  I would put her forward for promotion.  I would designate her as my proxy in my absence. |

*Note.*  English translation of the items has not been validated so far. Only the German items were part of the data collection.
